# Man’s Pursuit of Meaning: Unexpected Termination Bolsters One’s Autonomous Motivation in an Irrelevant Ensuing Activity

**DOI:** 10.3389/fnhum.2020.00081

**Published:** 2020-03-27

**Authors:** Wei Wei, Zan Mo, Jianhua Liu, Liang Meng

**Affiliations:** ^1^School of Management, Guangdong University of Technology, Guangzhou, China; ^2^Laboratory of Neuromanagement and Decision Neuroscience, Guangdong University of Technology, Guangzhou, China; ^3^School of Business and Management, Shanghai International Studies University, Shanghai, China; ^4^Wharton Neuroscience Initiative, The Wharton School, University of Pennsylvania, Philadelphia, PA, United States

**Keywords:** meaningfulness, fluid compensation, meaning maintenance model, autonomous motivation, reward positivity, event-related potentials

## Abstract

Meaningfulness has been suggested as one of the fundamental psychological needs, as one would actively pursue meaning in both his/her work life and personal life. Previous studies consistently showed that a lack of meaning in work would reduce one’s autonomous motivation in the current job, which is the motivation to engage in self-determined activities driven by one’s own interests or personal beliefs. However, researchers overlooked the fact that in work settings, it is not uncommon that people work on multiple tasks in a row. As a result, the cross-task effect of work meaningfulness remains understudied. Based on the meaning maintenance model (MMM) and the suggested fluid compensation strategy, we predicted that the disappearance of the meaning of work may induce a compensatory response and thus enhance one’s autonomous motivation in an irrelevant ensuing activity. To test this hypothesis, we invited participants to work on an encyclopedic knowledge quiz in Session 1 and a StopWatch (SW) task in Session 2. A between-subject design was adopted. While participants in the control group successfully completed their tasks in Session 1, those in the experimental group encountered unexpected program quits by the end of the quiz, and their previous efforts suddenly became futile and meaningless. Electroencephalography was recorded during the experiment to measure reward positivity (RewP). In Session 2, a more pronounced RewP in the win–lose difference wave was observed in the experimental group in contrast to the control group, suggesting that the disappearance of the meaning of work enhanced one’s autonomous motivation in an irrelevant activity that follows. Therefore, results of this study provided preliminary electrophysiological evidence for one’s pursuit of meaning and the compensation effect induced by the disappearance of the meaning of work.

## Introduction

As one of the most promising research topics in industrial and organizational psychology, meaningfulness is capturing more and more academic attention from researchers in the past few years. While the precise definition of meaningful work is still under debate, no one would deny the fact that it has become one of the most fundamental characteristics in the workplace. Findings of the classical “Lego experiment” well illustrated the importance of meaningfulness in the work setting. Despite the fact that the wage structure and the experimental task were identical in both conditions, when the experimenter disassembled each just-assembled Bionicle Lego model into pieces in front of the experimental subjects, the assembling task immediately became futile. As a result, these subjects significantly reduced their labor supply and assembled fewer models compared to their counterparts in the control condition, who could observe accumulation of the models they assembled (Ariely and Kamenica, 2008).

According to Ariely and Kamenica (2008), there are important prerequisites for meaningful work. For instance, in order to be perceived as meaningful, the work should have some point or purpose, which links one’s endeavors to some explicit or implicit objectives. The objective can be either personal or social-oriented (Martela and Pessi, 2018). The personal perspective is about self-realization. When an individual could get a sense of autonomy, authenticity, and self-expression at work, they would perceive the intrinsic value and thus the meaning of work (Martela and Riekki, 2018). The social perspective is about broader purpose. Once people realize that their jobs serve some greater good or prosocial goals (i.e., helping certain beneficiaries, including colleagues and customers), they would deem their work as significant and intrinsically worth doing (Grant, 2008; Allan et al., 2018; Martela and Pessi, 2018).

A growing body of literature has examined the antecedents and outcome variables of meaningful work in experimental settings (Ariely and Kamenica, 2008; Grant, 2008; Bäker and Mechtel, 2013; Chandler and Kapelner, 2013; Chadi et al., 2017; Kosfeld et al., 2017; Allan et al., 2018), and it was consistently found to be beneficial, both to the individuals and to the organization. Then, what if the meaning of work suddenly disappears? As far as we are concerned, only one pioneering study paid attention to the unexpected termination of a work project and explored its effects on the affected workers’ emotional responses and performances (Chadi et al., 2017). When a work project terminated unexpectedly, all the workers’ prior efforts suddenly became futile and the meaning of work disappeared. As a consequence, the workers were found to exert less effort in subsequent work tasks (Chadi et al., 2017). To conclude, a cross-task spillover effect was observed. This phenomenon in not uncommon in the workplace. In a widespread and influential TED talk, Dan Ariely, a renowned behavioral economist, told the audiences a true story that took place in a big software company in Seattle. When the chief executive officer (CEO) announced that a project got canceled, the 200 engineers working on this project in the past 2 years became depressed and less motivated. However, they told Dan Ariely in private that something could have been done by their CEO to convince them that their prior efforts were not totally meaningless.

As people would actively pursue meaning during their lives as well as work lives, meaningfulness has been suggested to be one of the fundamental human needs by a line of literature (Yeoman, 2014). Thus, given that a project has to be canceled anyway, wise employers could still provide the affected employees with an opportunity to restore their meaning of work in the next work project, which would minimize the side effects. After repeated deliberation, we consider that the spillover effect observed by Chadi et al. (2017) might be a result of the workers’ helplessness. Given that the preceding work project got terminated without a good reason, the workers might fear that history repeats itself; that is, the subsequent work project would be aborted as well. As a result, their work motivation got diminished and their work effort got reduced. In a recent organizational psychology study, boredom experienced in a previous task was found to enhance one’s performance on another task that follows (Shin and Grant, 2019). It appeared that people were engaged in the second task to a greater extent as a compensation for their lack of interest in the first task. In a similar manner, when unexpected termination happened, if people were reassured that the two work tasks were independent and irrelevant with each other, and that the unexpected termination was unlikely to take place again, then a compensation effect might be observed instead of a spillover effect. As the second task provides a person with an opportunity to pursue and restore the meaning of work, one’s autonomous motivation, the motivation to engage in self-determined activities driven by one’s own interests or personal beliefs (Deci and Ryan, 2000; Ryan and Deci, 2017; Fang et al., 2018) would get strengthened.

To test this “compensation” hypothesis, we conducted an experiment with a between-subject design. Participants in both groups (Experimental Group: experimental procedure unexpectedly terminated by the end of the first session; Control Group: participants completed both sessions without a termination) were instructed to work on an encyclopedic knowledge quiz (adapted from Wang et al., 2018) in Session 1 and a StopWatch (SW) task (adapted from Ma et al., 2014) in Session 2. It is worth noting that different experimenters carried out the two experimental sessions respectively, and that the two sessions were implemented in different experimental cubicles, minimizing the participants’ fear that an unexpected termination would happen again during Session 2. To examine the effect of the disappearance of meaning of work on one’s autonomous motivation in a following task, we recorded electroencephalograms (EEGs) of all participants throughout the experiment. Specifically, we resorted to reward positivity (RewP), a classical event-related potential (ERP) component observed during feedback processing and outcome evaluation to measure one’s autonomous motivation level (Ma et al., 2014; Meng and Ma, 2015; Fang et al., 2018, 2019).

Originally proposed by Holroyd and colleagues, RewP was named as feedback-related negativity (FRN) and was considered to be a frontal-central negative deflection in the past few decades (Hajcak et al., 2006; San Martin, 2012; Walsh and Anderson, 2012). Given that more and more empirical findings suggested that the negative deflection elicited by the negative feedback is just a baseline response, while it is the positive feedback that elicits a positive deflection (Ma et al., 2014; Proudfit, 2015; Mühlberger et al., 2017; Wang et al., 2018), a consensus on this ERP component has been reached. Nowadays, the RewP is commonly accepted as a positive deflection maximizing between 250 and 350 ms, which is more pronounced in response to positive outcomes as compared to negative ones (Distefano et al., 2018; Glazer et al., 2018; Hassall et al., 2018; Wang et al., 2018). According to the motivational significance theory, one of the predominant theories of RewP, the RewP in the win–lose difference wave (RewP in response to losses subtracted by that elicited by wins) responds to the motivational and/or affective influence of feedback information, whose magnitude represents a rapid subjective evaluation of the feedback’s motivational significance to the participants (Gehring and Willoughby, 2002; Yeung et al., 2005; Masaki et al., 2006). Previous literature consistently reported that a more pronounced RewP in the win–lose difference wave would be observed when the feedback is perceived to be more motivationally significant to the participants (Yeung et al., 2005; San Martin, 2012; Meng and Ma, 2015; Wang et al., 2018).

More relevant to the scope of the current study, in the past few years, a line of literature resorted to the win–lose difference wave of RewP to measure one’s autonomous motivation (Ma et al., 2014; Meng and Ma, 2015; Fang et al., 2018, 2019). In many neuroscientific investigations, performance-based monetary rewards are provided, and people pay much attention to their performance feedback. However, when external incentives and punishments no longer exist, and an individual participates in an activity in an autonomous manner, it was found that one’s dopaminergic value system still responds to informational feedback (Di Domenico and Ryan, 2017; Reeve and Lee, 2019). As a likely origin of RewP suggested by researchers (Tricomi et al., 2006; Depasque and Tricomi, 2015), the anterior striatum is deeply involved in reward processing and feedback evaluation. Thus, in a pioneering and influential functional magnetic resonance imaging (fMRI) study, Murayama et al. (2010) tracked activity in the anterior stratum and used it to measure one’s autonomous motivation when performance-based rewards were not provided. In line with this pioneering study, RewP in the form of win–lose difference wave was adopted to measure one’s autonomous motivation in existing literature (Ma et al., 2014; Meng and Ma, 2015; Fang et al., 2018, 2019) as well as the current study.

As the pursuit of meaning is within human nature, in this study, we predicted that the unexpected termination of a preceding task would induce a compensation effect in an irrelevant task that follows, which manifests as one’s strengthened autonomous motivation and engagement in the second task. Thus, we predicted to observe a more pronounced RewP in the win–lose difference wave in the experimental group compared to the control group. Besides, previous literature consistently suggested that individuals differ in their level of pursuit of meaning (Wrzesniewski et al., 1997; Ariely and Kamenica, 2008; Rosso et al., 2010; Lips-Wiersma and Wright, 2012; Allan et al., 2016; Chadi et al., 2017). Indeed, some would actively pursue the meaning of work, while others may not care about the meaning of work that much. Thus, we predicted that, after the meaning of work disappeared, those participants who had a greater pursuit of meaning would have enhanced autonomous motivation and/or engagement in a following task that provided the opportunity for meaning restoration.

## Materials and Methods

### Participants

Forty-eight undergraduate students from varied majors were recruited *via* advertisements at a university in southern China. Before participant recruitment, a power analysis was performed to estimate the appropriate sample size. We assumed the effect size (f) to be 0.4 and the error probability (α) to be 0.05, and the suggested sample size was 44. Thus, our sample size meets the requirement. Data from two participants were excluded due to insufficient valid trials after artifact rejection. Thus, there were 46 valid participants (24 females; ranging in age from 19 years to 23 years: *M* = 20.07, *SD* = 2.285). They received a compensation of 60 Chinese yuan (about 8 dollars) for participating in this study. All participants were healthy, right-handed, and had normal or corrected-to-normal vision. Nobody reported any medical, neurological, or psychiatric disorders. The participants were randomly assigned to the experimental (*N* = 23, 10 males) and the control group (*N* = 23, 12 males). This study was approved by the internal review board of the School of Management, Guangdong University of Technology. All participants provided written informed consent before the start of their experimental sessions.

### Experimental Paradigms

During the recruitment stage, participant candidates were informed that, once they sign up, they would be participating in two irrelevant experiments organized by different experimenters. While the duration of each experiment was rather short, the preparation and setup of an EEG experiment is quite time-consuming. Thus, the participants were led to believe that the two experimenters formed an alliance to conduct experiments together, while each of them was still fully responsible for his/her own experiment. The recruited participants who showed up at the laboratory were led to sit on a comfortable chair in a room which is dimly lit, sound-attenuated, and electrically shielded. Stimuli were displayed at the center of a computer monitor 100 cm away from the participants, with a visual angle of 8.69° × 6.52° (15.2 cm × 11.4 cm, width × height). Before the whole experiment started, the participants were told that they would receive 60 Chinese yuan (around 8 dollars) in compensation for their participation in the two experiments altogether. Therefore, their reimbursement was irrelevant to their task performances in either experiment.

**Figure 1:**
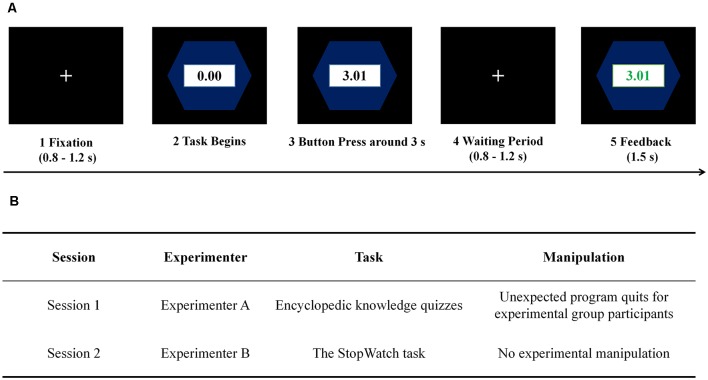
Demonstration of the experimental paradigm. **(A)** The StopWatch (SW) task. **(B)** The overall experimental procedure.

All participants were instructed to work on an encyclopedic knowledge quiz (adapted from Wang et al., 2018) in Session 1 and an SW task (adapted from Ma et al., 2014) in Session 2 (Figure 1B). To familiarize the participants with the experimental tasks, there was a practice stage at the beginning of each experimental session. For participants in the experimental group, we designed and implemented an unexpected termination of the experimental procedure by the end of Session 1. By that time, they should have completed 76–80 trials among a total of 80 trials. The participants were told that, because of the termination, all their experimental data went lost. Thus, the meaning of work suddenly disappeared. To minimize the participants’ fear that an unexpected termination would happen again during Session 2, they were convinced that this was due to an unknown systematic error, which was unlikely to happen again. In addition, the second experimenter led the participants to a different experimental cubicle to continue Session 2. It is worth noting that participants in the experimental group still received the proper payment. Participants in the control group completed Session 1 without a termination. As Session 1 only served as the experimental manipulation in this study, details of the encyclopedic knowledge quiz as well as the experimental data in Session 1 would not be reported here.

During Session 2, participants in both groups were instructed to complete 80 trials of the SW task of moderate difficulty. In this game, a watch would automatically start running, and participants should try their best to stop it around 3 s (Murayama et al., 2010; Albrecht et al., 2014; Ma et al., 2014; Meng et al., 2016). The closer, the better. The success interval in this game is 2.93 s-3.07 s. This time window was adopted in several pioneering literature which recruited comparable Chinese participants, which ensured that typical participants would succeed in approximately 50% trials (Fang et al., 2018, 2019). The within-trial procedure was shown in Figure 1A. Every trial started with a cross symbol, which lasted for 800–1,200 ms. After the watch symbol started running, participants needed to stay focused and to press the button so as to stop the watch within the designated interval. By the end of each trial, participants would be provided with the performance feedback that lasted for 1,500 ms. Feedback for wins would be displayed in green, and feedback for losses in red. A random blank interval lasting for 600–1,000 ms appeared on the screen before the next trial began.

After the two experimental sessions, the participants were asked to complete two questionnaires concerning their search for meaning and task engagement, respectively. The search for meaning scale was adapted from the scale developed by Steger et al. (2006), while the task engagement scale was adapted from the Utrecht work engagement scale (Schaufeli et al., 2006). The search for meaning scale measures one’s trait-level meaning pursuit tendency, which is relatively stable. In contrast, the task engagement scale measures one’s state-level engagement in a given task. It is worth pointing out that the task engagement scale includes three dimensions, which are motivation, energy, and focus. Both of them are 7-point scales, ranging from 1 (Do not fully agree) to 7 (Totally agree). Coding of the tasks, presentation of the stimuli, and recording of triggers and response are performed by E-Prime 2.0 (Psychology Software Tools, Pittsburgh, PA, USA).

### Electroencephalogram Recordings and Analyses

The EEG data were collected at a sampling rate of 500 Hz with Neuroscan Synamp2 Amplifier (Neurosoft Labs Inc., Sterling, VA, USA) using 64-channel standard electrodes placed on the participants’ scalp. In this study, we resorted to the magnitude of RewP to measure one’s autonomous motivation. Since existing studies showed that the amplitude of RewP is most pronounced at the central frontal electrodes, such as Fz and FCz (Ullsperger et al., 2014; Oemisch et al., 2017; Fernandes et al., 2018), in this experiment, EEG data were only collected at frontal electrodes (Fz, FCz, and Cz are the central electrodes among the selected ones).

The left mastoid served as an online reference, while the electrode on the cephalic region was set as ground. The electrooculogram (EOG) was recorded through four electrodes placed in the left and right orbital rim (horizontal EOG) and below and above the left eye (vertical EOG). Electrode impedance were kept below 10 kΩ throughout the experiment. During off-line data analyses, EEG data of each participant were processed by Letswave 6. These data were re-referenced, which used the average of the bilateral mastoids as the benchmark, after which the data went through a band-pass filter (between 0.5 and 30 Hz). Ocular artifacts caused by eye movements were corrected using the algorithm embedded in the Letswave program. For the RewP, time windows of 200 ms before and 800 ms after onset of the feedback were segmented, with the activity from −200 to 0 ms serving as the baseline. For each participant, EEG data over each recording site were averaged under each experimental condition. Trials containing amplifier clippings, bursts of electromyography activity, or peak-to-peak deflection that exceeded ±100 μV were excluded from statistical analyses.

Since the most positive peak of the win–lose difference wave of RewP occurs around 250 ms after onset of the feedback, the average voltage within 200–300 ms entered the analyses. In recent years, Luck and Gaspelin (2017) suggested that researchers should be cautious when including the electrode as an additional factor during ERP data analyses. Following this suggestion, an electrode cluster (F1, Fz, F2, FC1, FCz, FC2) was selected for the RewP analyses of this study.

## Results

### Behavioral Results

According to results of the independent sample *t*-test, there was no significant between-group differences in the success rates of the encyclopedic knowledge quiz [Success Rate_Experimental_ = 0.509 (*SD* = 0.078); Success Rate_Control_ = 0.519 (*SD* = 0.052); Cohen’s *d* = −0.015, *t*_(44)_ = 0.392, *p* = 0.697]. In the SW game, the mean error is defined as the absolute value of the difference between the responding time and the target time point (3 s). During Session 2, neither success rate [Success Rate_Experimental_ = 0.493 (*SD* = 0.053); Success Rate_Control_ = 0.492 (*SD* = 0.057); Cohen’s *d* = 0.018, *t*_(44)_ = 0.067, *p* = 0.947] nor mean error [Mean Error_Experimental_ = 0.1169 (*SD* = 0.012); Mean Error_Control_ = 0.1167 (*SD* = 0.009); Cohen’s *d* = −0.019, *t*_(44)_ = −0.005, *p* = 0.996] were significantly different.

A 2 (group: experimental group and control group) × 3 (engagement dimension: motivation, energy, and focus) mixed-model ANOVA was adopted in the analyses of participants’ responses to the task engagement scale. Group was a between-subject factor, while engagement dimension was a within-subject factor [Motivation_Experimental_ = 5.094 (*SD* = 0.668), Motivation_Control_ = 4.717 (*SD* = 0.829); Energy_Experimental_ = 5.254 (*SD* = 0.905), Energy_Control_ = 4.870 (*SD* = 0.773); Focus_Experimental_ = 5.449 (*SD* = 0.967), Focus_Control_ = 4.855 (*SD* = 0.864)]. Although participants in the experimental group had a greater task engagement than their counterparts in the control group on average, the main effect of group (*F*_(1,44)_ = 5.695, *p* = 0.436, *η*^2^ = 0.115) was not significant. Neither was the main effect of dimension (*F*_(1,44)_ = 0.286, *p* = 0.709, *η*^2^ = 0.006). The interaction effect between group and dimension was significant (*F*_(1,44)_ = 0.610, *p* = 0.032, *η*^2^ = 0.014). Results of simple effect analyses showed that the group effect was significant in none of the engagement dimensions [the motivation dimension: (*F*_(1,22)_ = 2.634, *p* = 0.112, *η*^2^ = 0.056), the energy dimension: (*F*_(1,22)_ = 0.737, *p* = 0.395, *η*^2^ = 0.016), the focus dimension: (*F*_(1,22)_ = 0.026, *p* = 0.874, *η*^2^ = 0.001)].

### Event-Related Potential Results

After EEG data preprocessing, the averaged trial numbers were Experimental Group-Win = 39.43 (*SD* = 4.219) and Experimental Group-Lose = 38.70 (*SD* = 3.611) in the experimental group, while these were Control Group-Win = 39.35 (*SD* = 4.529) and Control Group-Lose = 39.26 (*SD* = 3.958) in the control group. Results of the independent sample *t*-test showed that the numbers of successes (*t*_(44)_ = 0.193, *p* = 0.947, Cohen’s *d* = 0.018) and failures (*t*_(44)_ = −0.549, *p* = 0.568, Cohen’s *d* = 0.160) were not significantly different between the experimental group and the control group.

**Figure 2:**
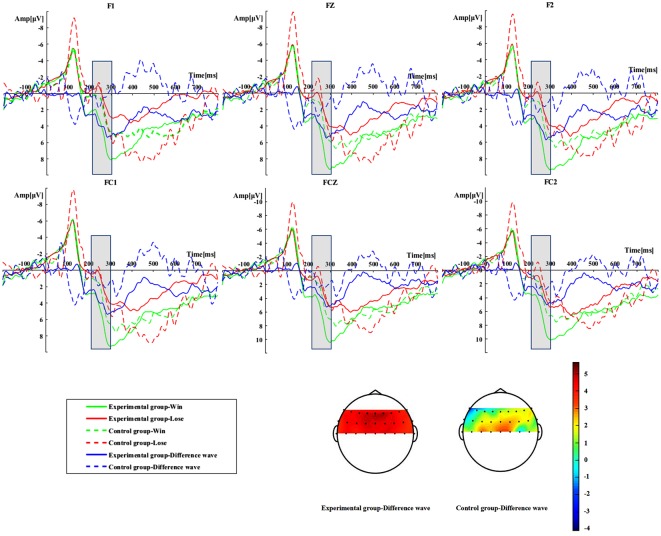
Reward positivity (RewP) results during outcome evaluation. Grand-averaged event-related potential (ERP) waveforms of RewP and its win–lose difference wave from the electrodes of F1, Fz, F2, FC1, FCz, and FC2 were shown for the group (experimental group vs. control group) and outcome (win vs. loss) conditions. The scalp topographic distribution of the win–lose difference wave of RewP is plotted for the recorded electrodes, and the bar ranges from −4 to 5 μV.

As demonstrated in Figure 2, the mean amplitudes of RewP were 8.0917 μV (experimental-win), 3.6489 μV (experimental-lose), 3.9437 μV (control-win), and 2.3007 μV (control-lose) in respective conditions. A two-factor mixed-model ANOVA analysis for the win–lose difference wave of RewP showed significant main effects of both the feedback valence (*F*_(1,44)_ = 39.83, *p* < 0.001, *η*^2^ = 0.475) and the group (*F*_(1,44)_ = 5.695, *p* = 0.021, *η*^2^ = 0.115). The interaction effect between group and feedback valence was significant as well (*F*_(1,44)_ = 8.430, *p* = 0.006, *η*^2^ = 0.161). This indicated that the amplitude of the win–lose difference wave of RewP in the experimental group (4.443 μV) was more pronounced than that in the control group (1.643 μV). Results of subsequent simple effect analyses showed that the feedback valence effects were significant in both the experimental group (*F*_(1,22)_ = 42.454, *p* < 0.001, *η*^2^ = 0.491) and the control group (*F*_(1,22)_ = 5.806, *p* = 0.02, *η*^2^ = 0.117). However, the group effect was found only in the RewP in response to positive feedbacks (*F*_(1,22)_ = 10.769, *p* = 0.02, *η*^2^ = 0.196), but not that elicited by negative ones (*F*_(1,22)_ = 1.197, *p* = 0.28, *η*^2^ = 0.026).

### Correlational Analyses

**Figure 3:**
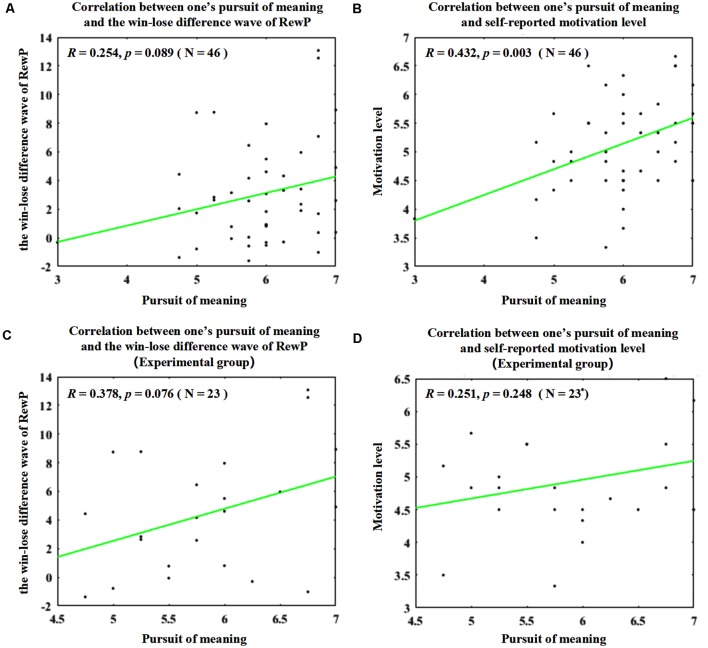
Scatter diagrams and results of the correlation analyses. **(A)** Correlation between one’s scores in the pursuit of meaning scale and the amplitudes of reward positivity (RewP) in the win–lose difference wave. **(B)** Significant correlation between one’s scores in the pursuit of meaning scale and the self-reported motivation level measured by the task engagement scale. **(C)** Correlation between one’s scores in the pursuit of meaning scale and the amplitudes of RewP in the win–lose difference wave (experimental group participants only). **(D)** Correlation between one’s scores in the pursuit of meaning scale and the self-reported motivation level measured by the task engagement scale (experimental group participants only).

To probe the effect of the pursuit of meaning as a potential individual difference factor (Figure 3), Pearson correlational analyses were conducted between one’s scores in the pursuit of meaning scale and the amplitudes of RewP in the win–lose difference wave (*r* = 0.254, *p* = 0.089; approaching statistical significance), as well as between one’s scores in the pursuit of meaning scale and the self-reported motivation level measured by the task engagement scale (*r* = 0.423, *p* = 0.003). When similar analyses were conducted on participants in the experimental group only, no significant results were found (correlation between one’s pursuit of meaning and the amplitude of RewP in the win–lose difference wave: *r* = 0.378, *p* = 0.076, approaching statistical significance; correlation between one’s pursuit of meaning and the self-reported motivation level measured by the task engagement scale: *r* = 0.251, *p* = 0.248).

## Discussion

While meaningful work is fundamental and beneficial both to the employees and to the organization, it is not uncommon that a work project got terminated and the meaning of work suddenly disappeared. In the work setting, people normally work on multiple tasks in a row. Thus, unexpected termination of a preceding task might have a non-negligible impact on one’s autonomous motivation in an ensuing task. To explore this effect in an experimental setting, participants were asked to work on two different tasks in two experimental sessions, respectively. In addition, a between-subject experimental design was adopted, and participants in the experimental group (but not the control group) encountered unexpected program quits by the end of Session 1. Success rates during Session 1 were comparable between the two groups, and no confounding factors were introduced to this study. We resorted to the magnitude of RewP in the win–lose difference wave to measure one’s autonomous motivation during Session 2 and observed a more pronounced RewP in the experimental group. This finding suggested that one has a pursuit of meaning, and that the disappearance of the meaning of work enhanced one’s autonomous motivation in an irrelevant ensuing activity. In addition, our correlational analyses results suggested that those participants who had a greater pursuit of meaning had enhanced motivation in the SW task.

In the vast majority of existing studies on human motivation, researchers have examined varied influencing factors of human motivation. However, a single task was adopted in most studies, neglecting the fact that a person may work on multiple tasks in a row, and that one’s experience and psychological state in a prior task may have a considerable impact on one’s motivation and engagement in a following task (Newton et al., 2020). In recent years, several pioneering studies found that frustration of the fundamental psychological needs would induce a restorative response (Radel et al., 2011, 2014; Fang et al., 2018, 2019). For instance, in an experiment with a two-session between-subject design, Fang et al., 2018 introduced competence frustration to participants in the experimental group and provided the opportunity of competence restoration to participants in both groups. Participants who experienced competence frustration beforehand were found to have a more pronounced RewP in the win–lose difference wave upon feedback during Session 2, suggesting that they had enhanced autonomous motivation in the second task (Fang et al., 2018). This finding provided the first empirical evidence for the competence restorative process. Compensation effects are ubiquitous. Findings of Fang et al. (2018, 2019) actually gave direct support for the compensation effect induced by competence frustration, which shared a similar psychological mechanism with findings of the current study.

Our findings can well be explained by the meaning maintenance model (MMM), according to which people have a need for meaning in a broader sense. Thus, all experiences and stimuli that violate the meaning system will evoke an aversive arousal, which in turn prompts individuals to perform a series of compensatory behaviors so as to alleviate negative emotions and restore meaning (Heine et al., 2006; Proulx and Inzlicht, 2012; Proulx et al., 2012). Fluid compensation is the key hypothesis in MMM. According to this hypothesis, when meaning is threatened in a certain area, individuals do not necessarily respond to this threat in a direct manner. It is possible that they try to reconstruct the meaning system by affirming and seeking meaning in other fields or domains. For participants in the experimental group, the task terminated unexpectedly, and the meaning of work disappeared. Since all the experimental data went lost, and they were not allowed to redo the experiment for time concern, the meaning of work could not be restored in the current experimental task. However, they could put their hearts and soul into the following experimental task so as to restore the meaning of participating in our experiments (Meng and Ouyang, 2020), which was exactly what we found in this study.

At first glance, findings of this study seemed to be contrary to those of the study conducted by Chadi et al. (2017). However, a major difference between the two studies is that in our study, participants in the experimental group were reassured that the two experimental tasks were irrelevant and that unexpected termination was highly unlikely to take place again in the second task. When this was the case, participants could take the opportunity to reconstruct their meaning systems in Session 2, and they had enhanced autonomous motivation as reflected by a greater win–lose difference wave of RewP. It is worth pointing out that similar patterns were not found in self-reported task engagement, that is, participants in the experimental group did not report themselves to be more engaged with the SW task compared to their counterparts in the control group. This might suggest that the compensation effect induced by the disappearance of meaning happens at the subconscious level. In other words, without knowing it themselves, participants who experienced the disappearance of meaning beforehand would try to pursue and restore the meaning of work in an irrelevant ensuing activity.

In this study, we also examined the pursuit of meaning as a potential individual difference factor during meaning pursuit and restoration. Indeed, we found that people differed in their levels of the pursuit of meaning, and that those who had a greater level of meaning pursuit reported to have enhanced motivation in the SW task. This finding suggested that while monetary rewards were widely believed by practitioners to be the greatest or even the only effective motivator in the workplace, actually one’s pursuit of meaning may work as another powerful motivating force, which contributes to one’s motivation and well-being (Kosfeld et al., 2017). However, it is worth noting that the correlational analyses results reached statistical significance only when data from both experimental group participants and control group participants were combined together. Thus, the role of the pursuit of meaning during meaning restoration still awaits further investigation in future studies. A potential limitation of this study is that EEG data were only collected at frontal electrodes. When conducting this experiment, we made this decision given that the amplitude of RewP was consistently reported to be most pronounced at the central frontal electrodes, such as Fz and FCz (Ullsperger et al., 2014; Oemisch et al., 2017; Fernandes et al., 2018). However, data should have been collected at all electrodes, which would allow for additional analyses.

The current study opens up new directions for future research on the meaning of work and meaning restoration. To pursue and restore meaning in an irrelevant task is just one of the possible restorative responses people may have. As an extension, researchers may explore other possible restorative responses to the disappearance of meaning. We believe that people’s restorative responses may vary, depending on both situational factors and individual difference factors. For instance, after experiencing disappearance of the original meaning, individuals may endow the work that they have accomplished with alternative meaning (e.g., self-fulfillment). In addition, while the unexpected termination of a work project is a small probability event, it is much more common that employees have to work on a meaningless work project in the first place. In this situation, a compensation effect induced by meaningless work may take place as well, as certain psychological and behavioral strategies (e.g., to work on something more meaningful) may be adopted to compensate for the insufficiency of meaning (Meng and Ouyang, 2020).

Findings of this study bear important practical implications for the managerial practice. In people’s daily jobs, there are situations when a work project gets canceled and the meaning of work disappears. For instance, different groups of people work on different work projects. These projects compete with each other. At a certain stage, only the project that wins over the others would go ahead and gets further support from the management. It is a pity that on most occasions, the employers fail to realize the gravity of this situation and decide to ignore such an event, which may undermine the employees’ enthusiasm in the next work project. As was pointed out by Chadi et al. (2017), when the meaning of work suddenly disappeared, the employers could help restore the employees’ perceived meaning of work. For instance, they may talk about the event in public and try to provide a reasonable explanation. They may even convince the employees that their previous efforts were not entirely wasted (Chadi et al., 2017). Our findings suggested that through providing the employees with an opportunity to restore meaning, the employers may provide a remedy for the loss of the meaning of work by working even harder in subsequent tasks.

## Conclusion

To explore the fluid compensation hypothesis proposed by the MMM, we designed a two-session experiment and assigned participants into the experimental group and the control group, respectively. Participants in both groups worked on an encyclopedic knowledge quiz in Session 1 and an SW task in Session 2. However, experimental group participants encountered unexpected program quits by the end of Session 1. These participants showed a more pronounced RewP in the win–lose difference wave upon feedback during Session 2 compared to their counterparts in the control group, suggesting that the disappearance of the meaning of work enhanced one’s autonomous motivation in an irrelevant ensuing activity. Through providing the first electrophysiological evidence for the fluid compensation strategy induced by the disappearance of the meaning of work, this study opens up new directions for follow-up research on the meaning of work and suggests practical guidelines for the managerial practice.

## Data Availability Statement

The datasets generated for this study are available on request to the corresponding author.

## Ethics Statement

This study was approved by the internal review board of the School of Management, Guangdong University of Technology. All participants provided written informed consent before the start of their experimental sessions.

## Author Contributions

LM conceived and designed the study and administered the project. WW and JL conducted the experiment and collected the data. WW analyzed the data. LM and WW interpreted the data and drafted the manuscript. LM, WW, ZM, and JL reviewed and edited the manuscript.

## Conflict of Interest

The authors declare that the research was conducted in the absence of any commercial or financial relationships that could be construed as a potential conflict of interest.
